# Differential thalamocortical interactions in slow and fast spindle generation: A computational model

**DOI:** 10.1371/journal.pone.0277772

**Published:** 2022-12-12

**Authors:** Muhammad Mushtaq, Lisa Marshall, Maxim Bazhenov, Matthias Mölle, Thomas Martinetz

**Affiliations:** 1 Institute for Neuro- and Bioinformatics, Lübeck, Germany; 2 Institute of Experimental and Clinical Pharmacology, University of Lübeck, Lübeck, Germany; 3 Center for Brain, Behavior and Metabolism, Lübeck, Germany; 4 University Clinic Hospital Schleswig Holstein, Lübeck, Germany; 5 Department of Medicine, University of California, San Diego, La Jolla, California, United States of America; Georgia State University, UNITED STATES

## Abstract

Cortical slow oscillations (SOs) and thalamocortical sleep spindles are two prominent EEG rhythms of slow wave sleep. These EEG rhythms play an essential role in memory consolidation. In humans, sleep spindles are categorized into slow spindles (8–12 Hz) and fast spindles (12–16 Hz), with different properties. Slow spindles that couple with the up-to-down phase of the SO require more experimental and computational investigation to disclose their origin, functional relevance and most importantly their relation with SOs regarding memory consolidation. To examine slow spindles, we propose a biophysical thalamocortical model with two independent thalamic networks (one for slow and the other for fast spindles). Our modeling results show that fast spindles lead to faster cortical cell firing, and subsequently increase the amplitude of the cortical local field potential (LFP) during the SO down-to-up phase. Slow spindles also facilitate cortical cell firing, but the response is slower, thereby increasing the cortical LFP amplitude later, at the SO up-to-down phase of the SO cycle. Neither the SO rhythm nor the duration of the SO down state is affected by slow spindle activity. Furthermore, at a more hyperpolarized membrane potential level of fast thalamic subnetwork cells, the activity of fast spindles decreases, while the slow spindles activity increases. Together, our model results suggest that slow spindles may facilitate the initiation of the following SO cycle, without however affecting expression of the SO Up and Down states.

## Introduction

Sleep plays an important role in memory consolidation. Two thalamo-cortical oscillatory rhythms, the sleep slow oscillations (SO) and sleep spindles of non-rapid eye movement (NREM) sleep play a comprehensive role in declarative memory consolidation [1–3]. It is believed that cortical SOs provide a temporal frame where recently-acquired memory can be replayed, and transferred to cortical regions for long-term memory storage [4–6]. During sleep spindles, which occur endogenously in temporal association with both the SO and hippocampal sharp wave ripples [7] processes of long-term potentiation and synaptic plasticity take place [8–10]. During the large amplitude SOs so-called Up states the majority of cortical neurons fire or become depolarized and maintain this depolarized state for several hundred milliseconds. Conversely, during the Down state, virtually all cortical neurons remain silent or exhibit a hyperpolarized state for a few hundred milliseconds [11–14].

In humans, sleep spindles are classified into two categories: fast spindles (12–16 Hz) and slow spindles (8–12 Hz), with differential properties. Fast spindles are well studied regarding their origin, generating mechanism, functional role, temporal, and spatial appearance in different cortical regions [15–18]. Slow spindles–mainly found in human deep sleep EEG–have been less extensively studied and their source, generation, and functional roles remain unclear. Some pharmacological and computational studies suggest that slow spindles may rely relatively more on cortical and less on thalamic sources as compared to fast spindles [19, 20]. On the other hand, the level of thalamic hyperpolarization has been suggested to affect differences in spindle frequency, and other spindle properties [21]. Bastuji et al. [22], using intrathalamic EEG recordings in humans, observed a significantly slower spindle frequency (11.89 Hz) in the ventral lateral posterior (VLp) thalamic nucleus as compared to other local posterior thalmic nuclei. Most interestingly the VLp was the most anterior of the recorded thalamic nuclei, and projects to the frontal cortical region.

In this study, we present a thalamocortical computational model for NREM sleep that exhibits both fast and slow spindles along with the SO rhythm. Fast and slow spindles are simulated in two independent fast and slow thalamocortical sub-networks, whereby each interacts with its own reticular sub-networks [23]. In the model both cell layers (TC and RE neurons) of the thalamic network for slow spindles are set to a more hyperpolarized level than the fast thalamic network. Our model provides a platform to investigate the interactive role of both fast and slow spindles during NREM sleep. Model results suggest that the role of slow spindles may be to facilitate ongoing cortical SO activity.

## Methods

**Fig 1:**
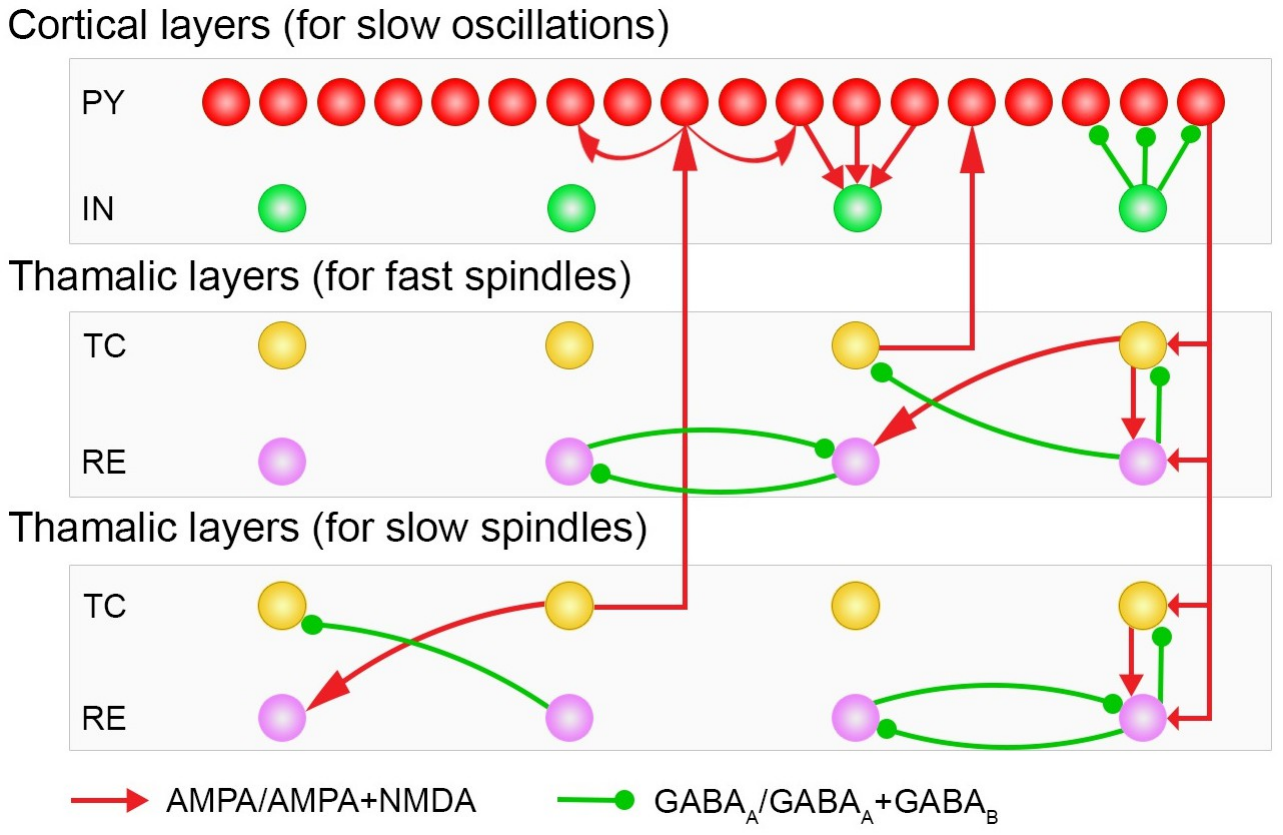
The thalamocortical network geometry and connection scheme. The network is comprised of six cell layers. The top two consist of cortical PY and IN neurons. The middle two layers are thalamic TC and RE neurons for fast spindle generation, and the bottom two thalamic TC and RE neuron layers generate slow spindles. The cortical PY cells layer contains 200 neurons, all other layers contain 40 neurons. Small green-filled circles symbolize GABA_A_Rs or GABA_A_Rs+ GABA_B_Rs, and corresponding connections. Red arrowheads represent correspondingly AMPARs or AMPARs+ NMDARs receptors.

In this study, a conductance based thalamocortical model for NREM sleep was extended from existing model [24]. All network neurons were modeled by Hodgkin-Huxley kinetics. Synaptic current calculations were based on first and higher-order synaptic activation schemes. Standard intrinsic currents and their conductance are described in Tables 1 and 2. The synaptic currents developed and balanced for SOs and both spindles are given in Table 3. Our thalamocortical network model incorporates three interactive sub-networks (Fig 1). The first is a cortical network for SOs comprising pyramidal cells (PY) and interneuron (IN). The second and third sub-thalamic networks are for fast and slow spindles, respectively. Each sub-thalamic network is comprised of a thalamocortical/relay (TC) and reticular (RE) cell layer.

**Table 1:** Dynamical models of all intrinsic currents.

**For PY neurons**
**(Axosomatic + dendritic) Fast sodium current *I***_**Na**_
*M* = 3; *N* = 1
α1 = 0.182(*V* + 25)/(1 − exp(−(*V* + 25)/9)) *if* |*V* − 10|/35 > 10^−6^ = 1.638 *if* |*V* − 10|/35 < 10^−6^
β1 = 0.124(−(*V* + 25))/(1 − exp((*V* + 25)/9)) *if* |*V* − 10|/35 > 10^−6^ = 1.116 *if* |*V* − 10|/35 < 10^−6^
τm = 0.34/(α1 + β1); *m*∞ = α1/(α1 + β1);
α2 = 0.024(*V* + 40)/(1 − exp(−(*V* + 40)/5)) *if* |*V* − 10|/50 > 10^−6^ = 0.12 *if* |*V* − 10|/50 < 10^−6^
β2 = 0.0091(*V* − 85)/(1 − exp(−(*V* − 85)/5)) *if* |*V* − 10|/50 > 10^−6^ = 0.0455 *if* |*V* − 10|/50 > 10^−6^
τh = (1/(α2 + β2))/2.9529; *h*∞ = 1/(1 + exp((*V* + 55)/6.2));
**(Axosomatic & dendritic) Fast potassium current *I***_**K**_
*M* = 1; *N* = 0;
α = 0.02 * (*V* − 25)/(1 − exp(−(*V* − 25)/9));
β = −0.002 * (*V* − 25)/(1 − exp((*V* − 25)/9));
τm = (1/(α + β))/2.9529; *m*∞ = α/(α + β);
**(Axosomatic + dendritic) Persistent sodium current *I***_**Na(p)**_
*M* = 1; *N* = 0;
*m*∞ = 0.02/(1 + *exp*(−(*v* + 42)/5));
**(Dendrite) Slow voltage-dependent non-inactivating potassium current *I***_**Km**_
*M* = 1; *N* = 0;
α = 0.001 × (*V* + 30)/(1 − exp(−(*V* + 30)/9));
β = −0.001 × (*V* + 30)/(1 − exp((*V* + 30)/9));
τm = (1/(α + β))/(−30); *m*∞ = α/(α + β);
**(Dendrite) Slow calcium-dependent potassium current *I***_**KCa**_
*M* = 1; *N* = 0;
α = 0.01 × [Ca^2+^]*i*;
β = 0.02;
τm = (1/(α + β))/2.9529; *m*∞ = α/(α + β);
**(Dendrite) High-threshold calcium current *I***_**HVA**_
*M* = 2; *N* = 1;
α1 = 0.055 × (−27 − *V*)/(exp((−27 − *V*)/3.8) − 1);
β1 = 0.94 × exp((−75 − *V*)/17);
τm = (1/(α1 + β1))/2.9529; *m*∞ = α1/(α1 + β1);
α2 = 0.000457 × exp((−13 − *V*)/50);
β2 = 0.0065/(exp((−*V* − 15)/28) + 1);
τh = (1/(α2 + β2))/2.9529; *h*∞ = α2/(α2 + β2);
**(Dendritic) Potassium leak current *I***_**KL**_
*M* = 0; *N* = 0;
*g*KL = 0.0025*mS*/*cm*^*2*^;
**For IN neurons**
IN cells have same current dynamics as PY cells except *I*_NA(p)_. *I*_NA(p)_ is not included in IN cells
**For TC neurons (TC neurons for both thalamic layers have same current dynamics)**
**Fast sodium current *I***_**Na**_
*M* = 3; *N* = 1;
α1 = 0.32 × (−37 − *v*)/(exp((13 − (*V* + 40))/4) − 1);
β1 = 0.28 × (*V* − 90)/(exp(((*V* + 40) − 40)/5) − 1);
τm = 1/(α1 + β1); *m*∞ = α1/(α1 + β1);
α2 = 0.128 × exp((17 − (*V* + 40))/18);
β2 = 4/(exp((40 − (*V* + 40))/5) + 1);
τh = 1/ (α2 + β2); *h*∞ = α2/(α2 + β2);
**Fast potassium current *I***_**K**_
*M* = 4; *N* = 0;
α1 = 0.032 × (−35 − *V*)/(exp((−35 − *V*)/5) − 1);
β1 = 0.5 × exp((−40 − *V*)/40);
τm = 1/(α1 + β1); *m*∞ = α1/(α1 + β1);
**Low-threshold calcium current *I***_**T**_
*M* = 4;*N* = 1;
*if V* < −63
τm = (1.0/(exp((*V* + 35.82)/19.69) + exp(−(*V* + 79.69)/12.7)) + 0.37)/3.9482; *m*∞ = 1.0/(1 + exp(−(*V* + 60)/8.5));
τh = 1.0/((exp((*V* + 46.05)/5) + exp(−(*V* + 238.4)/37.45)))/3.9482;
*if V* = −63
τh = 19.0/3.9482; *h*∞ = 1.0/(1 + exp((*V* + 78)/6));
**hyperpolarization-activated cation current *I***_**h**_
*Voltage dependence*: $C\overset{\alpha }{\to }O,O\overset{\beta }{\to }C$
*h*∞ = 1/(1 + exp((*V* + 75)/5.5));
τs = (20 + 1000/(exp((*V* + 71.5)/14.2) + exp(−(*V* + 89)/11.6)));
α = *h*∞/τs
β = (1 − *h*∞)/τs
***Calcium dynamics*:**
d[Ca]_i_/dt = -AI_T_ + ([Ca]_∞_—[Ca]i)/τ,
[Ca]_∞_ = 2.4 * 10^−4^ mM, *A =* 5.1819 * 10^−5^ mM cm^2^/(ms μA), τ = 5ms
**Potassium leak current *I***_**KL**_
*M* = 0; *N* = 0;
*g*KL = 0.03*mS*/*cm*^*2*^;
**For RE neurons (RE neurons for both thalamic layers have same current dynamics)**
**Fast sodium *I***_**Na**_ **and fast potassium current *I***_**K**_ (RE cells have same *I*_Na_ and *I*_K_ current dynamics as TC cells)
**Low-threshold calcium current *I***_ ***T*** _
*M* = 2; *N* = 1;
τm = (3 + 1/(exp((*V* + 27)/10) + exp(−(*V* + 102)/15)))/6.8986; *m*∞ = 1/(1 + exp(−(*V* + 52)/7.4));
τh = (85 + 1/(exp((*V* + 48)/4) + exp(−(*V* + 407)/50)))/3.7372; *h*∞ = 1/(1 + exp((*V* + 80)/5));
**Potassium leak current *I***_**KL**_
*M* = 0; *N* = 0;
*g*KL = 0.005*mS*/*cm*^*2*^;

**Table 2:** Model parameters, their values, and description.

Parameter name	Value	Description
**Cortical neurons, PY and IN (soma)**		
*C*_m_	.75 μF/cm^2^	Membrane capacitance
*g*_Na_	3000 mS/cm^2^ (PY; IN)	Maximal sodium conductance
*g*_K_	200 mS/cm^2^ (PY; IN)	Maximal potassium conductance
*g*_Na(p)_	15 mS/cm^2^ (PY)	Maximal persistent sodium
**Cortical neurons, PY and IN (dendrite)**		
*C*_m_	.75 μF/cm^2^	Membrane capacitance
*g*_Na_	1.5 mS/cm^2^ (PY; IN)	Maximal sodium conductance
*E*_Na_	50 mV (PY; IN)	Sodium reversal potential
*g*_KL_	0.003 mS/cm^2^ (PY; IN)	Potassium leakage conductance
*E*_LK_	-95 mV (PY; IN)	Potassium leakage reversal
*g*_L_	0.034 mS/cm^2^ (PY; IN)	Leakage conductance
*E*_L_	-68 mV (PY; IN)	Leakage reversal potential
*g*_Na(p)_	2.5 mS/cm^2^ (PY)	Maximal persistent sodium conductance
*g*_HVA_	0.01 mS/cm^2^ (PY; IN)	Maximal high-threshold Ca^2+^ conductance
*g*_KCa_	0.3 mS/cm^2^ (PY; IN)	Slow Ca^2+^ dependent K^+^ conductance
*g*_Km_	0.02 mS/cm^2^ (PY); 0.03 mS/cm^2^ (IN)	Slow voltage-dependent non-inactivating K^+^ conductance
**Thalamic neurons, TC and RE (for fast spindles)**		
*C*_m_	1 μF/cm^2^	Membrane capacitance
*g*_Na_	90 mS/cm^2^ (TC); 100 mS/cm^2^ (RE)	Maximal sodium conductance
*E*_Na_	50 mV (TC; RE)	Sodium reversal potential
*g*_K_	10 mS/cm^2^ (RE); 10 mS/cm^2^ (TC)	Maximal potassium conductance
*E*_*K*_	-95 mV (TC; RE)	Potassium reversal potential
*g*_KL_	0.033 mS/cm^2^ (TC); 0.005 mS/cm^2^ (RE)	Potassium leakage conductance
*E*_KL_	-95 mV (TC; RE)	Potassium leakage reversal potential
*g*_L_	0.01 mS/cm^2^ (TC); 0.05 mS/cm^2^ (RE)	Leakage conductance
*E*_L_	-70 mV (TC); -77 mV (RE)	Leakage reversal potential
*g*_T_	1.8 mS/cm^2^ (TC); 1.8 mS/cm^2^ (RE)	Low-threshold Ca^2+^ conductance
*g*_h_	0.025 mS/cm^2^ (TC)	Hyperpolarization-activated cation conductance
*E*_h_	-40 mV (TC)	Hyperpolarization-activated cation reversal potential
**Thalamic neurons, TC and RE (for slow spindles)**		
*g*_Na_	70 mS/cm^2^ (TC); 100 mS/cm^2^ (RE)	Maximal sodium conductance
*E*_Na_	50 mV (TC; RE)	Sodium reversal potential
*g*_K_	10 mS/cm^2^ (RE); 12 mS/cm^2^ (TC)	Maximal potassium conductance
*E*_*K*_	-95 mV (TC; RE)	Potassium reversal potential
*g*_KL_	0.03 mS/cm^2^ (TC); 0.015 mS/cm^2^ (RE)	Potassium leakage conductance
*E*_KL_	-95 mV (TC; RE)	Potassium leakage reversal potential
*g*_L_	0.01 mS/cm^2^ (TC); 0.016mS/cm^2^ (RE)	Leakage conductance
*E*_L_	-77mV (TC); -82 mV (RE)	Leakage reversal potential
*g*_T_	1 mS/cm^2^ (TC; RE)	Low-threshold Ca^2+^ conductance
*g*_h_	0.017 mS/cm^2^ (TC)	Hyperpolarization-activated cation conductance
*E*_h_	-40 mV (TC)	Hyperpolarization-activated cation reversal potential

**Table 3:** Synaptic receptors, conductance, and their connecting radii.

Source to target neuron	Receptor	Synaptic conductance (μS)	Connecting radius
**Intracortical connections**			
PY→ PY	AMPARs	.026	11
PY→PY	NMDARs	.0018	11
PY→IN	AMPARs	.05	3
PY→IN	NMDARs	.001	3
IN→PY	GABA_A_Rs	.16	11
**Intrathalamic connections (Fast spindles)**			
TC_(f)_→RE_(f)_	AMPARs	.025	17
RE_(f)_→TC_(f)_	GABA_A_Rs	.05	17
RE_(f)_→TC_(f)_	GABA_B_Rs	.01	17
RE_(f)_→RE_(f)_	GABA_A_Rs	.075	11
**Thalamocortical connections (Fast spindles)**			
TC_(f)_→PY	AMPARs	.012	21
TC_(f)_→IN	AMPARs	.012	5
**Cortico-thalamic connections (Fast spindles)**			
PY→TC_(f)_	AMPARs	.0013	21
PY→RE_(f)_	AMPARs	.0032	17
**Intrathalamic connections (Slow spindles)**			
TC_(S)_→RE_(S)_	AMPARs	.022	17
RE_(S)_→TC_(S)_	GABA_A_Rs	.22	17
RE_(S)_→TC_(S)_	GABA_B_Rs	.025	17
RE_(S)_→RE_(S)_	GABA_A_Rs	.05	11
**Thalamocortical connections (Slow spindles)**			
TC_(S)_→PY	AMPARs	.004	21
TC_(S)_→IN	AMPARs	.004	5
**Cortico-thalamic connections (Slow spindles)**			
PY→TC_(S)_	AMPARs	.0009	21
PY→RE_(S)_	AMPARs	.002	17

RE_(f)_ and TC_(f)_ thalamic neurons (Layer 3 and Layer 4 neurons) produce fast spindles and RE_(S)_ and TC_(S)_ thalamic neurons (Layer 5 and Layer 6 neurons) produce slow spindles.

### Cortical intrinsic currents

The pyramidal and interneuron cells of the cortex are modeled as two separate compartments (dendritic and axosomatic compartment) as initially proposed by Mainen and Sejnowski [25] based on Hodgkin-Huxley kinetics [24].


$$C_{m}\frac{dV_{D}}{dt}=-g_{L}\left(V_{D}-E_{L}\right)-g_{SD}\left(V_{D}-V_{S}\right)-I_{D}^{int}-I^{syn},$$

$$0=-g_{DS}\left(V_{S}-V_{D}\right)-I_{S}^{int}$$ (1)

In Eq 1, C_*m*_ and g_*L*_ are the membrane capacitance and leakage conductance of the dendritic compartment. E_*L*_ is the reversal potential, V_*D*_ the dendric and V_*S*_ is the axosomatic compartment membrane potential. g_*SD*_ and g_*DS*_ are the conductances between the axosomatic and dendritic compartments, respectively and g_SD = 1/(R*S_soma_ *165) and g_DS = 1/(R*S_soma_) where R = 10 MΩ and S_soma_ = 1.0*10^−6^ cm^2^. $I_{D}^{int}$ is the sum of active dendritic, $I_{S}^{int}$ the sum of active axosomatic currents and *I*^*syn*^ the sum of synaptic currents. $I_{D}^{int}$ and $I_{S}^{int}$ are the sum of the following intrinsic currents:

$$I_{D}^{int}=I_{Na}+I_{Na\left(p\right)}+I_{LK}+I_{HAV}+I_{Kca}+I_{Km},$$

$$I_{S}^{int}=I_{Na}+I_{Na\left(p\right)}+I_{K}.$$

Thereby, *I*_*Na*_ represents the fast sodium current, *I*_*Na(p)*_ the persistent sodium current, *I*_*LK*_ the potassium leak current, *I*_*HAV*_ the high-threshold calcium current, *I*_*Kca*_ the slow calcium-dependent potassium current, *I*_*KM*_ the slow voltage-dependent non-inactivating potassium current and *I*_*K*_ represents the delayed rectifier potassium current. The IN cell compartments have the same intrinsic currents except for *I*_*Na(p)*_ that is only included in PY cells, The ratio of dendritic area to somatic area was set to *ρ* = 165 in PY cells, and to *ρ* = 50 in IN cells. All voltage-dependent currents *I*_*c*_ were simulated in the same fashion:

$$I_{c}=g_{c}m^{M}h^{N}(V-E_{c})$$

where g_c_ is the maximum conductance, *m* is an activation gating variable, *M* is the number of activation gates, *h* is an inactivation gating variable, and *N* is the number of inactivation gates. *V* is the corresponding compartment voltage and *E*_*c*_ is the reversal potential. The dynamics of all gating variables were solved with the same equations:

$$\frac{dy}{dt}=-\frac{x-x_{\infty }}{\tau_{x}}$$

$$\tau_{x}=\left(1/\left(\alpha_{x}+\beta_{x}\right)\right)/Q_{T}$$

$$x_{\infty }=\alpha_{x}/\left(\alpha_{x}+\beta_{x}\right)$$

where x is a gating variable, *x* = *m* or *h*, the temperature-related term, *Q*_*T*_ = *Q* ^((T-32)/10)^ = 2.9529 where Q = 2.3, T = 36° C. *α*_*x*_ and *β*_*x*_ are voltage-dependent transition rates. All individual intrinsic currents are described in Table 1 and their units and parametric values are described in Table 2.

### Thalamic intrinsic currents

Two separate sub-thalamic networks for fast and slow spindles, respectively, were developed, each with a thalamocortical/relay (TC) and reticular (RE) cell layer. Cells of each layer were modeled based on a single compartment (somatic compartment) using the same voltage-dependent and calcium-dependent currents dynamics as expressed by Hodgkin-Huxley kinetics schemes [24]:

$$C_{m}\frac{dV}{dt}=-g_{L}\left(V-E_{L}\right)-I^{int}-I^{syn},$$ (2)

where *C*_*m*_ is the membrane capacitance, *g*_*L*_ the leakage conductance, *E*_*L*_ the reversal potential, and *V* the voltage of the compartment. *I*^*syn*^ denotes the sum of the synaptic currents and similarly *I*^*int*^ the sum of the active intrinsic currents. The sum of these active currents for TC, $I_{TC}^{int}$ and RE, $I_{RE}^{int}$ are described as

$$I_{TC}^{int}=I_{Na}+I_{k}+I_{KL}+I_{h}+I_{T},$$

$$I_{RE}^{int}=I_{Na}+I_{K}+I_{KL}+I_{T}.$$

here *I*_*Na*_ represents the fast sodium current, *I*_*K*_ the fast potassium current [26], *I*_*KL*_ the potassium leak current, *I*_*h*_ the hyperpolarization-activated cation current [27], *I*_*T*_ the low-threshold calcium current in TC [28] and *I*_*T*_ in RE neuron [29]. The potassium leak current is *I*_*KL*_ = *g*_*KL*_(*V-E*_*KL*_) in both TC and RE cells where *g*_*KL*_ is potassium leak conductance and *E*_*KL*_ is the potassium reversal potential (*E*_*KL*_ = -95 mV). Calcium dynamics for thalamic cells is described by;

$$\text{d}{\left[\text{Ca}\right]}_{\text{i}}/\text{dt}=-{\text{AI}}_{\text{T}}+\left({\left[\text{Ca}\right]}_{\infty }-\left[\text{Ca}\right]\text{i}\right)/\tau ,$$

where [Ca]_∞_ = 2.4 * 10^−4^ mM, *A =* 5.1819 * 10^−5^ mM cm^2^/(ms μA) and τ = 5ms.

All individual voltage-dependent currents were simulated in the same fashion as the cortical intrinsic currents given in Table 1. Table 2 gives the parametric values developed in our model.

### Synaptic currents

For synaptic signaling four types of synaptic currents, *I*_syn_ were used, three (AMPARs, GABA_A_Rs, and NMDARs) were modeled by the first-ordered activation scheme [30, 31]. Accordingly, these synaptic currents are given by

$$I_{syn}=g_{syn}\left[O\right]f\left(V\right)\left(V-E_{syn}\right)$$ (3)

where *g*_*syn*_ is the maximal synaptic conductance, [*O*] is the fraction of open channels and *E*_*syn*_ is the synaptic reversal potential. For AMPARs and NMDARs, *E*_*syn*_ = 0 mV, whereas for GABA_A_Rs, *E*_*syn*_ = -70 mV. For AMPARs and GABA_A_Rs *f*(*V*) = 1, for the NMDARs, the voltage-dependent sigmoidal function *f*(*V*) = 1/(1 + exp(−(*V* − *V*_*th*_)/*σ*)) was used [26, 30], where *σ* = 12.5 mV, *V*_*th*_ = -25 mV. The fraction of open channel [*O*] was computed by the following equation:

$$d\left[O\right]/dt=\alpha \left(1-\left[O\right]\right)\left[T\right]-\beta \left[O\right],$$

$$\left[T\right]=A\theta \left(t_{0}+t_{max}-t\right)\theta \left(t-t_{0}\right)$$

where *t*_*0*_ is the time for receptor activation and *θ*(*x*) is the Heaviside function [32]. The duration and amplitude parameters for the neurotransmitter pulse are *t*_*max*_ = 0.03 ms and A = 0.5. The synaptic current rate constants for AMPARs were *α* = 1.1 ms and *β* = 0.19 ms, for GABA_A_Rs *α* = 10.5 ms and *β* = 0.166 ms, and for NMDARs *α* = 1 ms and *β* = 0.0067 ms. Intracortical currents were modified by multiplying the short-term depression term “*D”* [33, 34] with the maximal synaptic conductance in Eq 3 for AMPARs and GABA_A_Rs receptors:

$$I_{syn}=Dg_{syn}\left[O\right]f\left(V\right)\left(V-E_{syn}\right),$$

where D is the amount of available synaptic resources, calculated by the following scheme:

$$D_{n+1}=1-\left(1-D_{n}\left(1-U\right)\right)\text{exp}\left(-\frac{\Delta t}{\tau }\right),$$

where the synaptic resources time recovery is *τ* = 700 ms, the interval between *nth* and (*n+1*) Δ*t*, and the fraction of resources used for each action potential is *U* (for AMPARs *U* = .07, for GABA_A_Rs *U* = .073).

The fourth synaptic current, GABA_B_Rs is computed by a higher-ordered activation scheme that involves potassium channel activation by a G-protein, [30, 35]:

$$I_{GABAB}=g_{GABAB}\left({\left[G\right]}^{4}/\left({\left[G\right]}^{4}+K\right)\right)/\left(V-E_{K}\right)$$

$$d\left[R\right]/dt=K_{1}\left(1-\left[R\right]\right)\left[T\right]-K_{2}$$

$$d\left[G\right]/dt=K_{3}\left[R\right]-K_{4}\left[G\right],$$

where [G] reflects the G-protein concentration, [R] the fraction of activated receptors, and the potassium reversal potential *E*_*K*_ = -95 mV. K1 = 0.052 m_M_^-1^ms^-1^, K2 = 0.0013 ms^-1^, K3 = 0.098 ms^-1^, and k4 = 100μ_M_^4^ were the rate constants. The maximal synaptic conductance used here for each synapse is described in Table 3.

### Network geometry

The network model is comprised of six one-dimensional layers of neurons (Fig 1). Each layer of cells has N neurons, (N = 40) except the PY neurons layer, which has 5N neurons (200 neurons) [36]. The first and second cortical layer of PY and IN neurons initiate SOs. The third and fourth are thalamic layers for fast spindle initiation, and similarly the fifth and sixth are also thalamic layers that initiate slow spindles. The radii of synaptic connections between different layers are described in Table 3. For each SO cycle initiation, EPSPs and IPSPs miniature currents were implemented to PY-PY, PY-IN and IN-PY cells via AMPARs and GABA_A_Rs receptors [37]. These mini currents emerge ~ 100 ms after the start of the SO downstate. For Poisson input implementation, NetStim.noise was set to 1 in the NEURON simulator.

### Computational environment

The model was simulated in the NEURON 7.6 simulation environment [38] and it was run on a MacBook Pro 2015. For data analysis, MATLAB (R2020a) and eeglab tool were used.

## Results

In this study, we developed a conductance based thalamocortical model for NREM sleep. As far as we know this is the first model for normal NREM sleep including fast and slow spindles written in the NEURON simulation environment. Our model exhibits both fast and slow spindles along with SOs. Both fast and slow spindles were initiated in two separate thalamic subnetworks. The main network comprises six layers of cells (Fig 1), the top two of which are cortical layers of PY and IN cells for SOs, while the middle two are thalamic layers of TC and RE cells that initiate the fast spindles (‘fast thalamic subnetwork’) and the lower two layers are for slow spindles (‘slow thalamic subnetwork’). The thalamic subnetwork for slow spindles has the same type of intrinsic and synaptic current dynamics and network architecture as the fast thalamic subnetwork, however, it has a larger hyperpolarization than the fast thalamic subnetwork. Hyperpolarization of the slow thalamic subnetwork was increased by setting the reversal potential of GABA_A_Rs to E_GABAA_ = -88 mV in TC cells. The reversal potential of passive currents was set to E_L_ = -77 mV in TC cells and E_L_ = -82 mV in RE cells.

### Model initiation of SO and spindle generation

**Fig 2:**
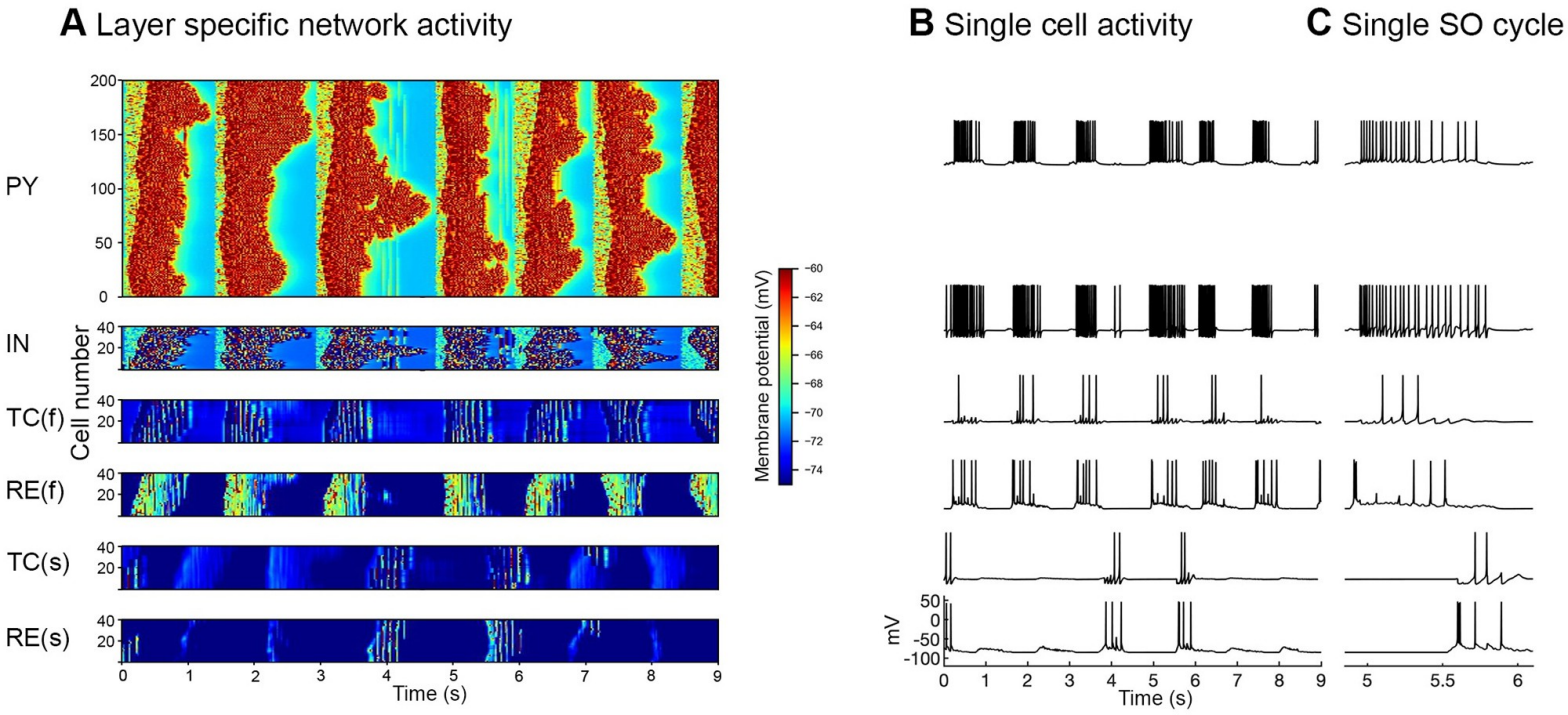
Layer specific network activity. ***A***, Space-time raster plots show the simultaneous activity of each neuron layer (200 PY, 40 IN, and 40 cells in each thalamic layer). The membrane potential of each cell is color coded. TC(f) and RE(f) represent thalamocortical and reticular cells of the fast thalamic network and similarly TC(s) and RE(s) represent thalamocortical and reticular cells of the slow thalamic network. ***B***, Corresponding single-cell activity of each neuron layer. Slow thalamic cells respond relatively seldom (bottom two) compared to the cells of the fast thalamic network. ***C***, Zoomed LFP and single cell activity of one SO cycle.

**Fig 3:**
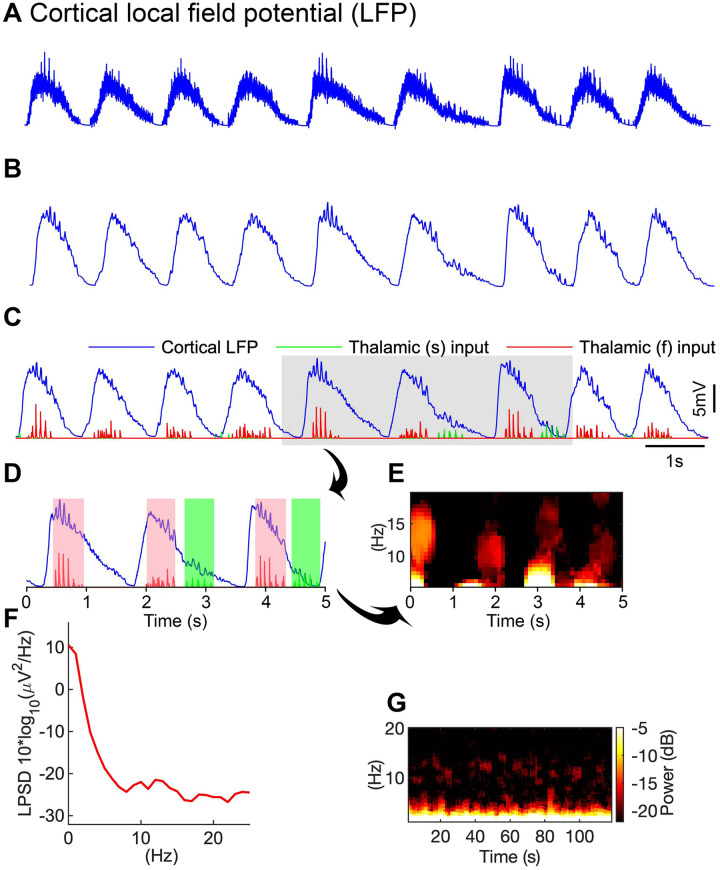
Resultant cortical local field potential (LFP). ***A***, Cortical LFP calculated as the sum of presynaptic currents (AMPARs, NMDARs, GABA_A_Rs) of PY cells. ***B***, Smoothed LFP revealing up and down SO states more clearly. ***C***, Spontaneously occurring Cortical LFP indicating times of both thalamic synaptic inputs. The occurrence of slow and fast spindles varies during long time simulations. The ‘fast thalamic’ input (red) responsible for fast spindle generation occurs at the SO down-to-up state transition SO. The slow thalamic input (green) is responsible for slow spindle generation and occurs at the SO up-to-down state transition. ***D***, zoomed figure of C indicating fast thalamic spindle (red) input nested within the first SO-half (pink filled box, time period of ~500 ms). The green input and green filled box describe slow thalamic input and its time period. ***E***, time-frequency spectrogram (calculated by short-time moving window Fourier transform) (range, 5–20 Hz) of LFP, for shaded area (panel E) indicates the bands of fast and slow spindles. ***F***, Log power spectrum of the LFP across the time period of 120 s, exhibiting power in the SO (~1 Hz), slow spindle (8–12 Hz), and fast spindle (12–16 Hz) frequency bands. ***G***, time-frequency spectrogram (range 0–25 Hz) of the LFP of 120 seconds duration indicates SO, fast and slow spindle activity.

The main thalamocortical network is initiated by mini synaptic current to both cortical layers during the hyperpolarized Down state. Once the SO cycle is initiated, the mini synaptic current is terminated. During the Down state, the mini synaptic current activates the persistent sodium current of PY neurons and consequently these PY neurons depolarize and reach firing threshold. Initially only one or few PY neurons generate an action potential, yet they target their neighboring PYs by strong PY-PY excitatory connections. Due to the strong PY-PY excitatory connections and persistent sodium current they sustain this depolarized Up state for 500–1000 ms. The calcium dependent potassium current and progressive synaptic depression terminate the depolarized Up state and bring cortical network back to the Down state. After ~100 ms of terminating the SO cycle, the mini synaptic current is again activated for the next SO cycle initiation and similarly this process is repeated for each SO cycle. Here we discuss the sequential flow of our network model after the activation of the cortical network. As the cortical network is activated, both layers of the fast thalamic subnetwork (TC and RE cells layers) also receive cortical inputs and become active. In the fast thalamic network, the interaction of TC and RE cells produces fast spindle oscillations with a major contribution of the TC hyperpolarization current (I_*h*_) and transient calcium current (I_*T*_) [39]. The fast thalamic output is sent back to the cortical network by TC cells. The cortical network receives this fast thalamic feedback ~200 ms after the initiation of the SO cycle (Fig 2). The thalamic network for slow spindles receives cortical inputs with a 600 ms delay. We specifically chose this delay of 600 ms after the initiation of SO to achieve the generation of slow spindles during the second SO-half. The netcon class of the NEURON simulation environment was used to model the delay. Hyperpolarization of the slow thalamic network was increased by setting the reversal potential of GABA_A_Rs to E_GABAA_ = -88 mV in TC cells. The reversal potential of passive currents was set to E_L_ = -77 mV in TC cells and E_L_ = -82 mV in RE cells. This more hyperpolarized thalamic network produced slow spindle oscillations that send excitation back to the cortical network via TC cells. The cortical network receives this slow thalamic input ~800 ms after the initiation of the SO cycle (Fig 2 bottom two raster plots of entire slow thalamic network activity, left, and unit activity, right). Cortical LFP was calculated as the sum of the presynaptic currents (AMPARs, NMDARs, GABA_A_Rs) of PY cells. The resultant cortical local field potential (LFP) is depicted in Fig 3A.

The LFP was smoothed using the function; filter() (MATLAB) with a window size of 200 for better visualization of spindles, nested with the cortical LFP (Fig 3B). The fast thalamic input (Fig 3C and 3D, red) projected to the cortical network ~200 ms after the initiation of the SO cycle, whereas the slow thalamic input (Fig 3C and 3D, green) projected to the cortical network ~800 ms after the initiation of the SO cycle, nearly a few hundred ms before the termination of the SO cycle. Similarly, the fast thalamic inputs were found in every SO cycle but the weaker inputs did not have an impact on the cortical LFP (Fig 3D 2^nd^ to 4^th^ SO cycle).

**Fig 4:**
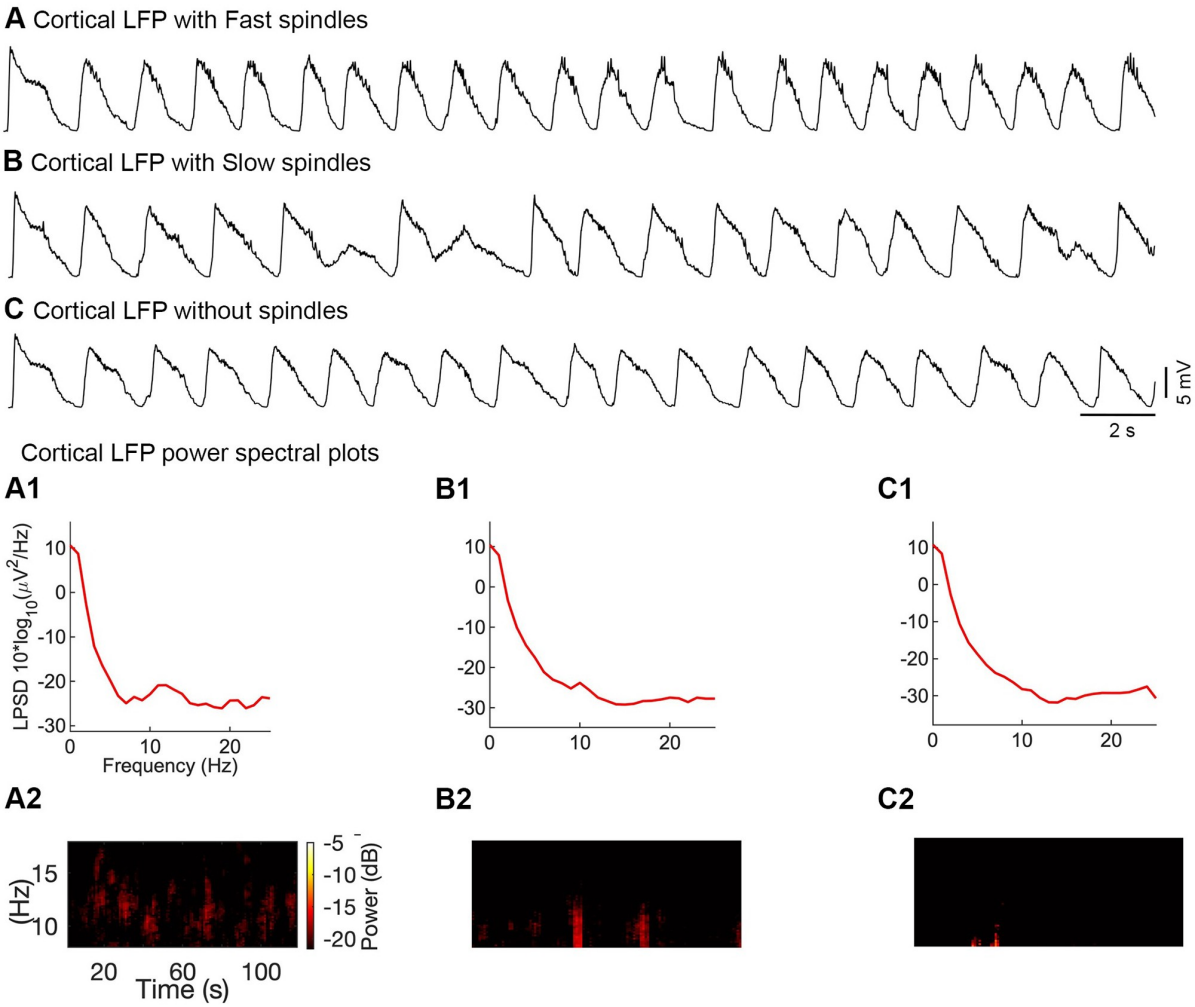
The influence of different thalamic inputs on the cortical LFP. ***A***, The cortical LFP coupled with fast thalamic spindles (slow spindles were blocked). Slow thalamic network layers (layers 5 and 6) were blocked by setting synaptic conductance to zero between PY-TC(S) and PY-RE(S) cells. ***A1***, Power spectra of the cortical LFP with fast thalamic spindles ***A2***, The corresponding time-frequency spectrogram of cortical LFP. ***B***, The cortical LFP with slow spindles (fast spindles were blocked). ***B1***, The corresponding power spectra of LFP. ***B2***, The corresponding time-fraency spectrogram of LFP. In the spectrum, the number of slow spindles was relatively low compared to fast spindles (See ***A2***). ***C***, The cortical LFP without fast and slow thalamic inputs. Fast and slow spindles disappeared in both the power (***C1***) and time-frequency (***C2***) spectrum.

**Fig 5:**
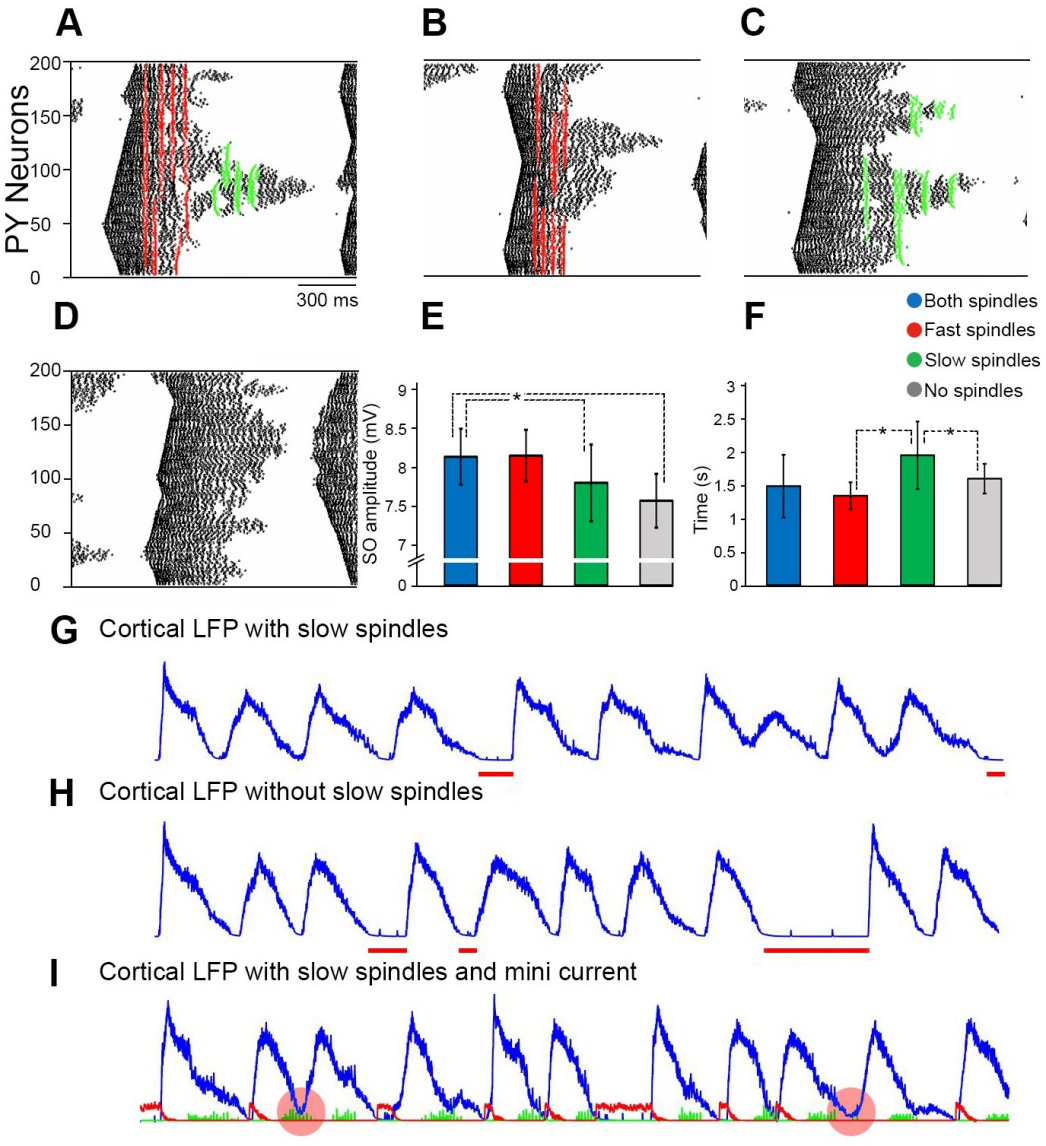
The influence of different thalamic inputs on the SO cycle dynamics. ***A*,** PY spike raster plots for one SO cycle with both fast and slow spindles inputs; ***B***, with only fast spindles (slow spindles were blocked). PY firing rate is relatively increased by fast spindle input (red); ***C***, with only slow spindles (fast spindles were blocked). PY firing rate is relatively increase by slow spindle input (green). ***D***, without spindle input (both fast and slow spindles were blocked). ***E***, Average cortical LFP amplitude in the presence of both fast and slow spindles (blue), fast spindles only (red), slow spindles only (green), and without spindles (gray). Average SO amplitude is lowest when spindles are absent. Asterisks show significant difference between the amplitudes (two sample t-test with *P < .01). ***F***, Average SO cycle duration dependent upon presence of spindles. Duration was longest for the presence of slow spindles. Error bars describe standard deviation between SO cycles. Asterisks show significant difference between the SO cycle duration (*P < .01). ***G*,** Resultant cortical LFP when the input of miniature synaptic current was reduced, but slow spindles were still generated by the model. The horizontal red bars indicate periods of increased down state duration due to weak mini synaptic current. ***H*,** Same as for G, however slow spindles are blocked. The down state durations are on average longer than when slow spindles are present (G). ***I***, The cortical LFP with the input of miniature synaptic current (red) and slow spindles (green). The red circles indicate SO initiated by slow spindles without mini current.

Moreover, in the temporal window the thalamic inputs with a lifetime of less than 0.5 seconds were not considered proper spindles. In normal simulations, the number of fast spindles was high compared to that of slow spindles (see Fig 3C above). In our slow spindles results, generally the waning phase of the slow spindle was completed before the completion of the SO cycle, although sometimes a few spikes of the waning phase were also observed after the completion of the SO cycle, i.e. in the down state of the SO. Sometimes slow spindles initiated the second SO cycle before the completion of the preceding SO cycle (Fig 4B 6^th^ and 8^th^ SO cycle). Slow spindles initiated approximately 10% of SO cycles during normal or control simulation (See Fig 5I below).

For further investigation, mini synaptic currents that initiate SOs, were reduced to observe the role of the slow spindle in SOs initiation. The time period of SO down states was shorter in the presence of slow spindles (see Fig 5G–5I). The finding that slow spindles may contribute to maintenance of SO activity is the major finding of our model.

Independence of fast and slow spindle networks was investigated in another set of simulations in which network activity was produced after blocking the fast thalamic network (blocking layer 3 and 4), slow thalamic network (blocking layer 5 and 6) or by blocking both thalamic networks (blocking all four thalamic layers). Results indeed show the temporal properties of fast and slow spindles can be retained independently of one another (Fig 4). The thalamic layers were blocked by setting the synaptic conductance to zero between the cortical PY cells and thalamic cells (PY to TC and PY to RE cells).

### Properties of SO—Spindle interactions

In-vivo fast spindles occur normally during the SO down-to-up transition and SO up state. We refer to this interval as the SO first-half in our model. Correspondingly, the second SO-half (~ 800 ms after the initiation of the SO) characterizes the up-to-down state transition during which slow spindles occur. The fast thalamic subnetwork input projected to the cortical network increases the firing rate of cortical cells (Fig 5A and 5B). Their spiking occurred earlier as compared to spikes from non-thalamically innervated cortical cells (Fig 5D). Furthermore, the first SO-half associated with fast spindles obtained a larger amplitude (Fig 5E) than without input from the fast spindle thalamic network. The average amplitude of SO cycles coupled with fast spindles was ~8.1 mV, whereas the average amplitude without spindles was ~7.6 mV. Thus, in our model, fast spindles increase both the firing rate of cortical cells and SO amplitude. Slow spindles emerge later, during the second SO-half around ~700 ms after the initiation of the SO cycle, when the SO has already reached its peak amplitude and starts to decline. Omission of the slow spindle thalamic subnetwork (Fig 5C vs. 5D) had two effects: a slowing of up state cortical firing rate, and a reduced SO duration. On the other hand, the duration of the second SO-half was more frequently longer in the presence of slow spindles than when only fast spindles were present (Fig 5C vs. 5A). Thus, in our model slow spindles appear to essentially assist in initiating the SO. To underpin this finding, we observed the model output across a time span of 120 second. The total time spent in SO down states was only ~16 seconds when slow spindles coupled to the SO up-to-down transition in the cortical LFP whereas without slow spindles a longer total time was spent in the down state, i.e. ~ 32.6 seconds. In this control simulation approximately 10% of SO cycles were initiated by slow spindles (e.g., Fig 5I red circled SOs): In cases of SO initiation by slow spindles miniature synaptic currents were not required for the initiation of SO.

**Fig 6:**
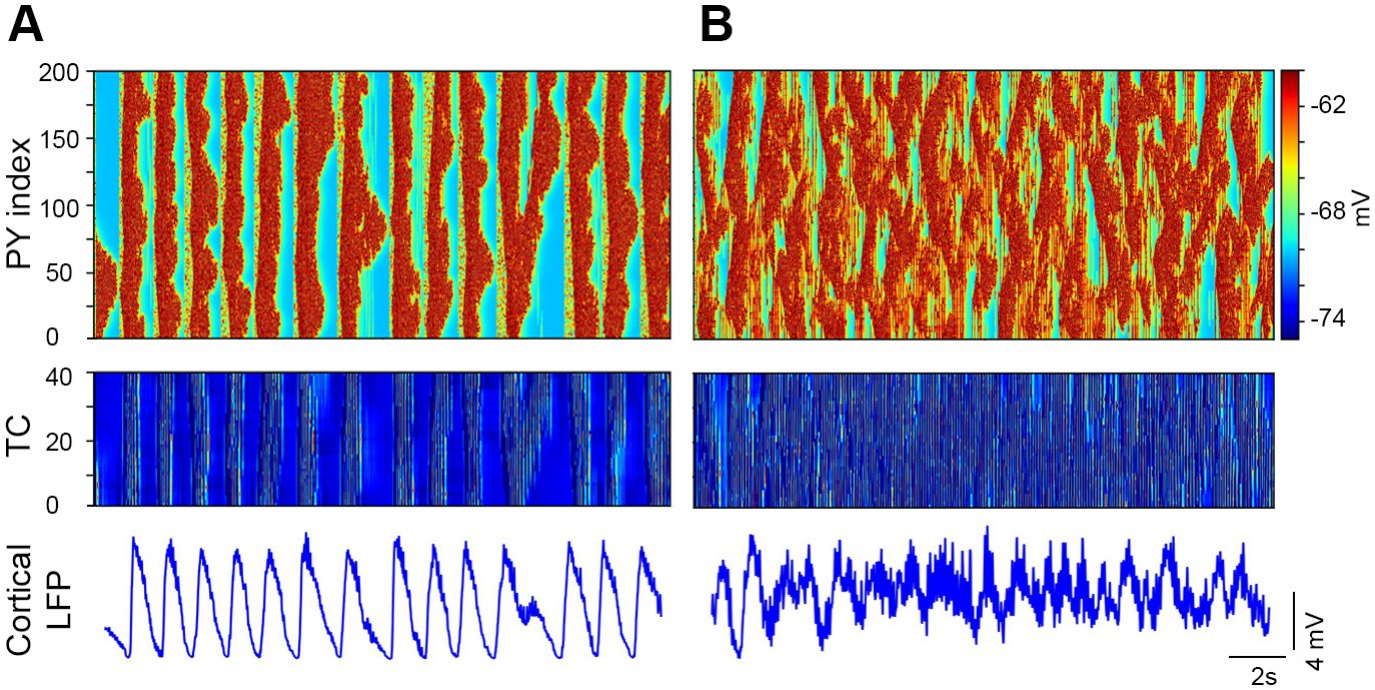
The influence of fast spindles in the SO cycle. ***A***, The control simulation in which the fast spindles are coupled with SO-first-half and slow spindles with the SO-second-half. Cortical and network exhibit regular LFP and both the up and down states of SOs are quite clear. ***B***, The simulation in which fast spindles were coupled with both phases of the SO cycle, the first half and the second half. Top panel: PY raster plot revealing that fast spindle coupling in the second phase almost diminished the down/hyperpolarized state of SO. Middle and bottom panels: Fast thalamic activity in both SO halves produced irregular (middle) and more depolarized (bottom) responses.

In the second-SO-half, the cortical network unit frequency is comprehensively reduced and cortical cells cease firing. To better distinguish between the interaction of slow and fast spindle subnetworks with SO properties in this critical second SO-half, we replaced slow spindles with the fast spindles (see Fig 6), i.e., fast spindles were generated both during the first and second SO-halves (Fig 6B). Fast spindles in the second SO-half eliminated the down/hyperpolarized state of SO, the cortical network remained in its state of increased firing (similar to the first SO-half), albeit exhibited activity was irregular compared to controlled simulation results (Fig 6A). In another control simulation, the model only allowed for generation of fast spindles during the second SO-half. The down states of SOs again disappeared and inconsistent cortical LFPs were observed (not shown in figure). From these results, we conclude that slow spindles maintain a slow oscillation rhythm by preserving the SO down state.

**Fig 7:**
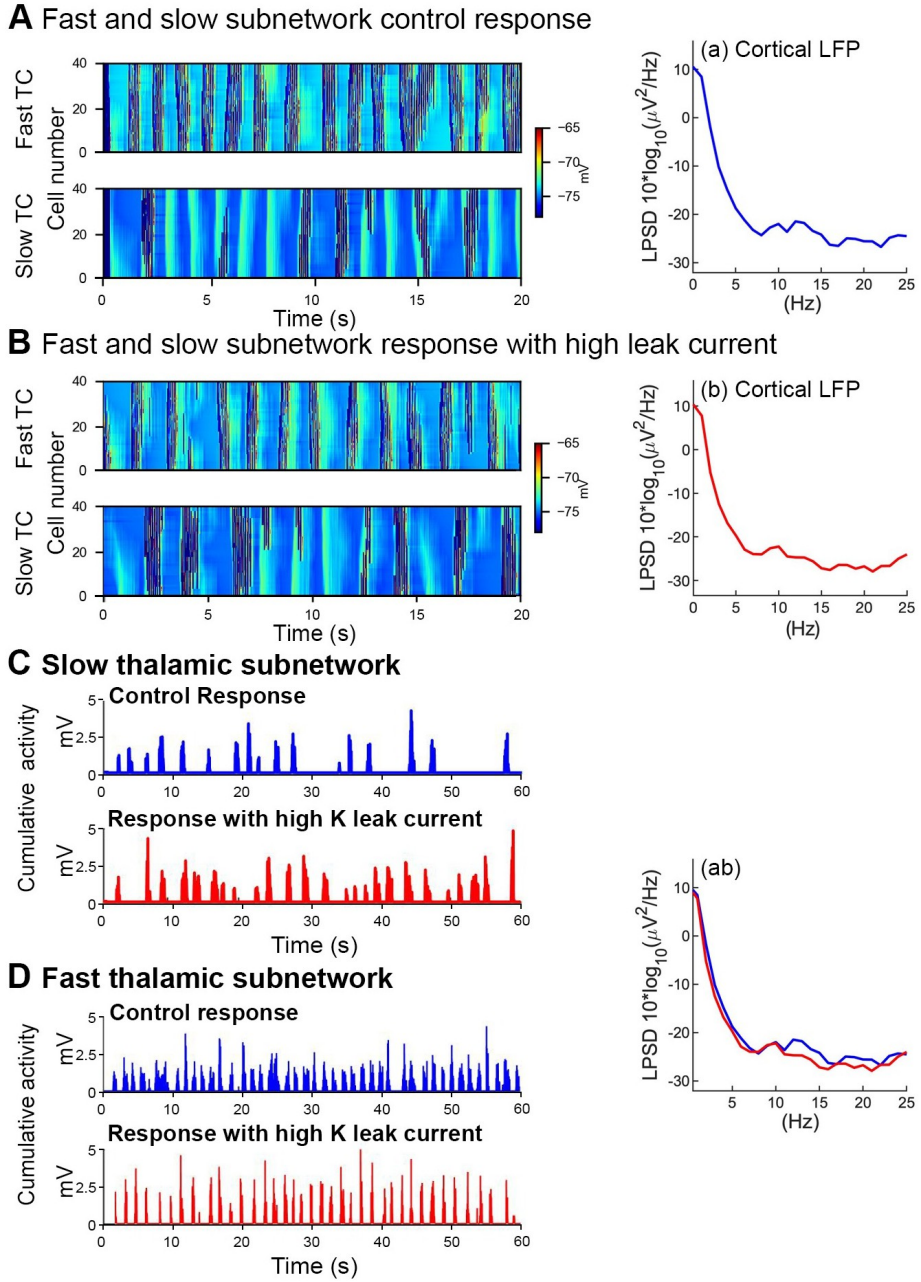
Influence of increased hyperpolarization of fast TC and increased potassium leak current in the fast thalamic subnetwork on spindle activity. ***A***, Plots of fast TC and slow TC cell membrane potential (color coded, right axis) overlayed by the firing rate (left axis) during control simulation (left) and the corresponding power spectrum of cortical LFP (right; a). ***B***, Plots of fast TC and slow TC cell membrane potential (color coded, right axis) overlayed by the firing rate (left axis) during hyperpolarization simulation (left) and the corresponding power spectrum of cortical LFP (right; b). ***C***, In control simulations, the input from the slow thalamic subnetwork projecting to the cortical network generated only a few events (~19 events per minute; above panel, left), whereas in simulations with increased K leak current, the number of events was higher (~25 events per minute; bottom panel, left). ***D***, In control results, The fast thalamic subnetwork exhibited stronger response in each event (~ 9 spikes in event; above panel) in control results, whereas in increased K leak these results, the event response was weaker (~ 6 spikes in event; bottom panel). ***ab***, The resultant cortical LFP power in the slow frequency range remained the same after hyperpolarization.

The thalamic membrane potential level (in both TC and RE cell layers) differs, physiologically, between depths of NREM sleep and thus plays a role in the likelihood of spindle generation [40, 41]. To investigate the response of the model, we increased the hyperpolarization level of fast thalamic subnetwork. Hyperpolarization was increased by altering the conductance of potassium leaked current (*g*_KL_): g_KL_ = .033 mS/cm^2^ (from *g*_KL_ = .03 mS/cm^2^). The hyperpolarization response is depicted by raster plots of the all TC cells in the fast (40 TC cells) and slow (40 TC cells) thalamic subnetworks and by the power spectra of the corresponding cortical LFPs (Fig 7): The membrane potential of TC cells of the fast thalamic subnetwork decreased with increased thalamic hyperpolarization compared to control fast thalamic subnetwork activity (Fig 7A and 7B). In each fast thalamic subnetwork with hyperpolarization a decreased number of spikes per event (~ 6 spikes) were observed relative to control simulations (~ 9 spikes/event; Fig 7D). Fast spindle LFP power also decreased with hyperpolarization (Fig 7B, left). Interestingly, this increase in hyperpolarization of the fast thalamic subnetwork resulted in a stronger slow thalamic subnetwork input to the cortical network and subsequently, the number of slow thalamic subnetwork events was increased (~25 events per minutes) as compared to the control simulations (~ 19 events per minute: Fig 7C). The resultant cortical LFP power in the slow frequency range remained the same after hyperpolarization (Fig 7ab right). In summary, the hyperpolarization of the fast thalamic subnetwork reduces fast spindle activity and increases activity within the slow thalamic subnetwork.

**Fig 8:**
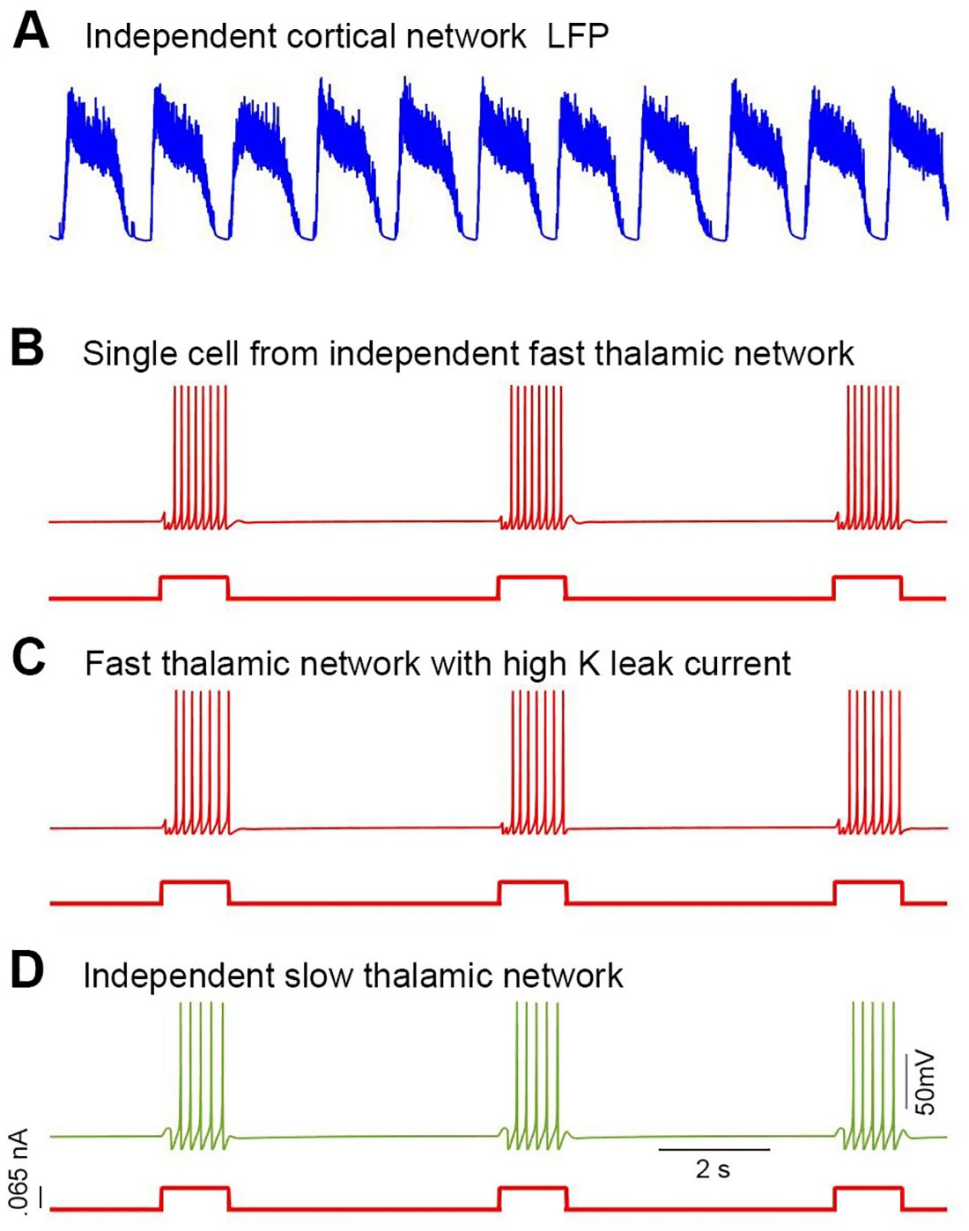
Each individual network response. **A**, The resultant local field potential of the individual (two layered) cortical network. ***B***, Single TC cell exhibits frequency ~16 Hz in individual fast thalamic subnetwork when step current was applied. ***C***, The TC cell of fast thalamic subnetwork with high potassium leak current shows relatively weaker response (~14 Hz). ***D***, The TC cell of slow thalamic subnetwork shows slow frequency ~10Hz.

Finally, we disintegrated all three subnetworks; cortical, fast thalamic, and slow thalamic subnetworks to validate the main rhythms; SO, fast spindles and slow spindles in each individual network. The cortical network initiation procedure was just like in the integrated model. Both fast and slow thalamic subnetworks were initiated by injecting step current to TC and RE cells. In both thalamic subnetworks, RE cells got .09 nA current whereas TC cells got .065 nA current for 600 ms after every 3 seconds (see Fig 8). All three networks successfully generated expected frequency range. Cortical network generated LFP with little high frequency ~ 1.3 Hz (Fig 8A). Thalamic results were observed in single cell activity. TC cells of the fast thalamic subnetwork fires with frequency of ~ 16 Hz. Moreover, the fast thalamic subnetwork shows weaker response with high potassium leak current (~ 14 Hz) (Fig 8B and 8C). Similarly, the slow thalamic subnetwork cells fire with frequency ~ 10 Hz (Fig 8D).

In summary, in our model slow spindle properties can be modeled by a thalamic subnetwork that differs from the fast spindle thalamic subnetwork; Our model revealed the following properties of SO and spindle interactions:

1. fast spindles increased the SO amplitude;
2. slow spindles assisted in initiating the SO;
3. slow spindles regularized and stabilized the SO rhythm by preserving the SO down state; and
4. hyperpolarization of the fast thalamic subnetwork reduced fast spindle density and increased the number of slow thalamic network events (slow spindles).

## Discussion

Sleep spindles are the other prominent EEG rhythm of NREM sleep. These are initiated by the interaction of thalamocortical (TC) and reticular (RE) cells of thalamic nuclei [39]. These spindles are also sent to the cortical network by collaterals of TC cells. Generally, these are nested with cortical SOs at the initial phase of the SO cycle, the first-half phase in primates and rodents. In human sleep studies, spindles are classified into two categories, namely fast (12–16 Hz) and slow (8–12 Hz) spindles. The fast spindles are mostly found in central and parietal cortical regions, and these are also nested with SOs during the first-half of up state. Slow spindles are observed in the frontal cortical area, and these are nested with SO during the second-half of up state, [18, 42]. Fast spindles are extensively studied in the experimental and computational arena regarding their role in long-term potentiation and synaptic plasticity [8]. Conversely, slow spindles need lot of work to find clear answers about their origin, cellular mechanism, and most importantly their role in such a framework (SO, fast spindles, hippocampal ripples) that is dedicated for memory consolidation. As a step ahead, it would be useful to explore the functional relation between SOs and slow spindles.

In this study, we developed a thalamocortical model for SOs, fast and slow sleep spindles. The novel aspect is the inclusion of thalamic-based slow spindle generation. Two independent sub-thalamic networks were developed for each fast and slow spindles. Physiologically fast and slow spindles occur at the SO down-to-up transition / SO up-phase and at the SO up-to-down transition, respectively. In the model fast spindles are nested with SOs during the first-half of the SOs cycle (SO down-to-up transition / SO up-phase) and the slow spindles are nested with SOs during the second SO-half (end of the SO up state/up-to-down transition). Slow spindles are produced with the same intrinsic and synaptic current dynamics as fast spindles but the thalamic subnetwork is in a more hyperpolarized state. According to our model results, slow spindles can initiate SOs and may thereby facilitate the maintenance of ongoing SOs. A contribution of slow spindles to the maintenance of activity in the SO state could also be deduced from the decrease in SO duration on omission of slow spindles. Faster electrophysiological activity ensues as NREM sleep lightens.

Fast spindles could not replicate these actions of slow spindles on the SO. When simulations were run for fast spindles nesting within the second SO-half (end of the up-state), the cortical network exhibited an irregular LFP pattern and the down states of SOs practically disappeared. Moreover, the fast thalamic subnetwork response was reduced when the hyperpolarization level in this network was increased, in contrast to the facilitatory response of the slow thalamic subnetwork to hyperpolarization. Thus, our model shows that slow spindles can facilitate cortical network activity while maintaining the natural rhythm of SOs.

To which neurophysiological processes are the model properties consistent? Prominent initial studies revealed that SO arise from layer 5 pyramidal cells and that SOs continue despite thalamic deafferentiation [43] e.g., through intrinsic activity emerging in layer 5 pyramidal cells [11, 43, 44]. It was recognized that thalamic input can contribute to cortical Up-states [45], however, the argument that SOs do not require thalamic input for initiation contrasts more recent studies that disclosed thalamic activity preceding the onset of cortical Up states, and also that severing thalamocortical connections reduced the incidence of spontaneous cortical Up states [46], and also modified ongoing SO frequency [47]. Experiments on anaesthetized cats demonstrated that thalamic oscillations contribute importantly to the cortical network in generating SOs, and can results explain previous contradictory findings [48], reviewed in [49]. Thalamic output at the time of slow spindle activity may thus deliver the input to the cortex required for triggering cortical Up states. Some thalamocortical cells, including the ventral lateral posterior (VLp) nucleus as a possible thalamic source of slow frequency spindles [22], possess both core-like and matrix neurons, and project thus to both superficial and deeper cortical layers, including axonal arborizations to layer V [50–52]. The intrinsic initiation of SOs in cortical layer 5 is discussed in the context of one the of two possible mechanisms, firstly, by persistently active pacemaker-like cortical cells, and secondly by temporal summation of spontaneous synaptic activity [44, 49]. Any one of both mechanisms cannot initiate the SOs unless it counters the activity-dependent K^+^ conductances that are activated during active states. Consistent with our modeling results slow spindle could assist in SO initiation by providing some depolarization to counter activity-dependent hyperpolarization conductances. Independent on whether such bilayer cortical input could be beneficial for SO initiation in layer 5 pyramidal cells, the above cytoarchitectonics and neurophysiological processes may allow to explain model results on slow spindle-SO initiation.

Processes underlying the maintenance of the SO rhythm are undoubtedly even more diverse and complex than can be reflected in our model. Human intracranial recordings and rodent local field potentials describe mechanisms on how thalamic spindles may drive cortical spindles emerging during the SO down-to-up transition [53, 54]. Slow and fast spindles are found however to occur during the same SO phase [53], thus findings on the temporal relationship between thalamic and cortical down states are not comparable with our modeling results. These inconsistencies in slow spindle timing present a major focus of ongoing research [55–57].

Modeling results presented two properties regarding the impact of fast spindle on the slow spindle network. Firstly, that fast spindles increased the firing rate of cortical cells and SO amplitude, and secondly that the hyperpolarization of the fast thalamic network reduced fast spindle, yet increased slow spindle activity. Both properties are associated with membrane potential level of thalamic cells, and coincide with results linking increased thalamic hyperpolarization to the emergence of delta oscillations and increased NREM sleep depth [58]. In deeper NREM sleep occurrence and frequency of spindles are also reduced in humans [21].

Taken together, our modeling results present above most a further platform to test the potential role to thalamic spindles in SO initiation. In future work, we will extend this study by making structural and physiological modifications for more detailed experiments. For instance, by including neuronal plasticity in the current model we could analzye the potential contribution of slow spindles to this function.
